# The Hospital Nursing Supplement

**Published:** 1895-09-07

**Authors:** 


"The Hospital" Nursing Mirror.

Being the Extra Nursing Supplement of "The Hospital" Newspaper.

[Contributions for this Supplement should be addressed to the Editor, The Hospital, 428, Strand, London, W.C., and should have the word "Nursing" plainly written in left-hand top corner of the envelope.]

### News from the Nursing World.

### THE HOME OR THE WORKHOUSE.

The importance of district nursing in relieving the pressure upon the workhouses, and lessening the demand for expensive infirmary treatment, was illustrated by a letter read at a meeting of the Hackney Board of Guardians from the secretary of the Haggerston and Hoxton District Nursing Association. This letter was an application for a subscription to their funds, and in it were enclosed the names and addresses of twenty-nine patients nursed by the association in Hackney, many of whom would otherwise have become chargeable to the union. After consideration of this letter, and in view of the work being done among the poor of the district, it was resolved that a donation of £5 5s. be made to the funds of the association, subject to the sanction of the Local Government Board.

### A STORM IN A TEAPOT.

We have received from Mrs. Currie, of Dennistown, Glasgow, a correspondence between her and Mrs. M. R. Nichol, secretary of the Midwives' Institute and Trained Nurses' Club, in regard, apparently, to an application by the former for admission to the institute. There seems to us no doubt that there was considerable delay on the part of the secretary in answering some of the letters, and perhaps a little more effort to be conciliatory in her forms of speech than was necessary in answer to the distinctly aggressive letters which she had received. On the other hand, it is not easy to learn from the correspondence why Mrs. Currie should have felt so irritated at being asked for a reference. We presume an institute for midwives and a club for trained nurses contains women only, and it does not seem a very out of the way proceeding to ask for a reference to a matron or other ladies rather than to doctors. It is clear that angry passions rose as soon as a communication was received from the secretary declining to accept printed testimonials as sufficient. Which was very silly.

### HOW SMALL-POX PATIENTS ARE NURSED.

The Asylums Board is very reticent as to all its nursing arrangements, it being impossible to gather from its published reports any details either as to the number or the training of the nurses employed at the great hospitals under its care. This is a matter of considerable public interest, and we think that the smouldering uneasiness which is continually showing itself, in one quarter or another, in regard to the internal management of these institutions will not be lessened by an advertisement which appeared in a morning paper on August 19th, and ran as follows: "Wanted, at once, at the small-pox hospital ships, Long Reach, near Dartford, second assistant nurses (no training necessary)," &c. Now, in the name of all that is reasonable, we would ask why is no training necessary for the nursing of small-pox? From the nurses' point of view, of course, the employment of untrained women for such a task as that of nursing patients suffering from such a disease as small-pox is utterly indefensible, and nurses may well ask what is the inducement to undergo a long and expensive training if untrained women are to be employed as nurses by a great public body such as the Asylums Board, and are thus to be allowed after a few months, or perhaps weeks, of work to earn the right to pose before the public as "hospital nurses." Obviously a great wrong is thus done to women who elect to undergo a proper training for their profession. It is, however, rather from the public point of view that we prefer to regard the matter. The recent spread of small-pox in the metropolis has made it increasingly clear that if the disease is to be kept at bay the most careful isolation must be practised, which means, in the great majority of cases, early removal to hospital. People may then well ask the question by whom they are to be nursed, and for whose benefit these untrained women are engaged? If they are really only engaged as probationers, and if the intention is to train them as fever or small-pox nurses, this should surely be stated in the advertisement. As it stands, this advertisement is not only calculated to create a very uneasy feeling in th mind of the public, but is sure to greatly increase the difficulty which the Board doubtless experiences in ob taining good nurses; for it is hardly to be expected that properly trained women will be anxious to enter a service where they may be classed along with those for whom no training is considered necessary.

### A GUARDIAN'S TESTIMONY.

At a meeting of the Bradford Board of Guardians held last month, in moving that the minutes of the Nursing Staff Committee—which included a recommendation to appoint seven new nurses—be adopted, Mr. Guy said that the provision of a proper number of nurses had been found to be the cheapest course in the long run, as the patients were in the hospital for a shorter period, and it was much more humane. The minutes were adopted.

### A HOLIDAY IN BED.

The *Daily Telegraph* makes merry over a story it has come across anent a couple of hospital nurses who adopted a novel method of enjoying their fortnight's holiday. They seem to have hired a cottage in the country, and an old woman to attend on them. But from the moment they entered the door until the time came for their departure they were never seen, and the villagers concluded there was some deep mystery connected with them, and even thought of consulting the police on the subject. On the way back to the station, however, they called on the vicar and explained the secret. They had spent their holiday in bed! After the broken rest and the long and weary standing they had been accustomed to the ideal holiday to them took the form of a long unbroken rest. This little story has a chestnut flavour, and no doubt is capable of considerable variations at the hands of a good *raconteur*. At the same time it verges so nearly on common sense that one must not altogether despise its moral, for it often is the case, and not for nurses only, that a couple of days in bed is a far better restorative than the tiresome trail to the seaside so many people indulge in.

### A HARD-HEARTED NURSE.

The following point has been put before us for solution, and we think it is a matter of considerable interest, and one which may well be considered carefully by our readers:—

A district nurse in a somewhat isolated country district would be very grateful if her fellow nurses would kindly give her their opinion in the following case. Doubtless, few of the outside world have the least idea what a real holiday means to a nurse—may I say, more especially to a country district nurse, whose work is necessarily more or less monotonous. The nurse in question, with the sanction of her committee, started one afternoon, in company with another nurse friend, prepared to thoroughly enjoy her three weeks' holiday, which she had requested instead of a month, as allowed by the rules, fearing the work might suffer from so long an absence. After a most enjoyable twelve days (funds not permitting of a longer change), she and her friend returned to her cottage to spend the remainder of her three weeks in peace and quietness. Imagine her surprise to find a note from her lady superintendent requesting her to visit a certain patient as soon as possible who lived some miles away, and who had been ill, but was getting better under the care of the local doctor. Having had experience in other districts, town and country, the nurse knew how difficult and unpractical it would be to favour one person without resuming her whole duty. After duly thinking the matter over, she thought it best to refuse to go until the expiration of her holiday, consequently she is thought to be very hard-hearted and unfeeling. This hard-hearted district nurse would be glad to know if she was justified in acting thus, and would be obliged if one or two of her fellow-nurses will tell her how they would have acted under the same circumstances.

A Hard-hearted Nurse.

To this profound ethical problem we would suggest that it seems quite impossible for anyone to take a holiday in the midst of work staring one in the face and crying aloud for attention. The attempt to spend the holiday at home seems to have been in this case the initial blunder. Consider a clergyman, a doctor, or a lawyer: not one of them could obtain rest by merely saying that, as it was his holiday, he declined to see anyone on business. Equally so the nurse. However much we may agree in theory with the proposition that the nurse may take her holiday in her own cottage, and put out of consideration for a given term of days the claims of the sick around her, we fear—nay, we think—that in practice this is impossible.

### PEEPING TOM!

There is no doubt that sometimes the guardians themselves are a difficulty in regard to the proper management of a workhouse infirmary. Surprise visits, and the right of the guardians to enter the workhouse at any reasonable time, give to the paupers a great protection, and we should be the last to say a word that should lessen the efficiency of these bulwarks against officialism. Nevertheless, it is impossible to be blind to the difficulties which may arise sometimes, so long as guardians remain the sort of men they too often seem to be at present. From the *News of the World* we gather that an investigation has recently been made by the Infirmary Committee of the Wandsworth and Clapham Union into statements made concerning two guardians, who are alleged to have been seen on the roof of the workhouse infirmary in company with three friends, close to the skylights which surmount the dressing and sleeping apartments of the night nurses. This, the committee ascertained, occurred on Sunday afternoon, and complaints were made by officials of the infirmary. The two guardians did not deny that they made a tour of the infirmary roof on the day in question, but it was to investigate the painting work that was being carried on there by a contractor, and their friends were experts. It was pointed out that they were infringing the regulations, and they replied that they had a right to do as they liked. Asked if it were true that they peeped through into the nurses' bedrooms, one of the guardians replied that "it was impossible to see anything, as it was ground glass." He was not ashamed of what he did or what he saw. He had a right to peep about on Sunday if he liked, and he should do it again, whatever the guardians or the Local Government Board said. It was stated that the night nurses were probably all in their apartments when the two guardians were on the roof. Ultimately the matter was left to be dealt with by the whole Board. There may be nothing in all this; but it becomes clearer every year that, considering the large number of women in poor law institutions—either as inmates, attendants, or nurses—women should be elected in a larger proportion than at present is the case upon the Boards of Guardians by whom these institutions are managed.

### HOME NURSING IN STOCKPORT.

Sick nursing at home is becoming an interesting matter to the people dwelling in the large manufacturing towns in the North of England, and it is pleasant to see such purely popular bodies as the friendly societies organising fêtes and processions with the object of supporting the nursing bodies. A few Sundays ago, with the object of raising funds for the Sick Nursing Association, the brethren of the United Order of Free Gardeners of Stockport inaugurated a grand church parade. The Order numbers some 1,300 members, but owing to unfavourable elements only about 400 joined in the procession. These, however, made a good show. The assembling took place in the Armoury, and, accompanied by four bands, who had willingly proffered their services, the Gardeners wended their way to the parish church, where an address was delivered by the Rector (Rev. W. Symonds). The effort resulted in the sum of over £53 being raised for the Sick Nursing Association, a most welcome addition to the funds, and doubly welcome as showing the appreciation in which the nurses are held.

### A CURIOUS QUESTION.

The *Parish Councillor* asks a pertinent question. "How comes it to pass," it asks, "that the Devonport Guardians have spent six months' salary of a nurse in advertising for one without receiving a single application for the situation? Has the amount of offered salary anything to do with it? How much do the Guardians offer, and what are the duties? Surely the enthusiasm for nursing is not extinguished!" It must be better to spend money on nurses than on advertisements.

### Elementary Physiology for Nurses.

By C. F. Marshall, M.D., B.Sc., F.R.C.S.

### V.—THE CIRCULATORY SYSTEM (*continued*).

### Coagulation of the Blood.

Coagulation, or clotting, is a most important property of the blood. If blood is drawn into a basin, at the time of drawing it is quite fluid, but in two or three minutes it becomes viscid, and in five or ten minutes a complete jelly, so that the basin containing it can be turned upside down without spilling it. A few minutes later drops of a thin yellowish fluid—serum—appear on the top, and increase rapidly, the clot meanwhile shrinking. In an hour the clot is floating on the surface of the serum. The clot consists of a fine network of interlacing fibres formed by a substance called fibrin. In the meshes of this network are the corpuscles. The corpuscles are, however, not essential to the clot, for if coagulation be retarded by cold or by chemical means the corpuscles will sink to the bottom before coagulation occurs. If we whip blood with a bundle of twigs, the fibrin separates in long, stringy threads, leaving the serum and corpuscles behind. Hence the fibrin which forms the clot is derived from the plasma, and plasma consists of fibrin and serum. The actual nature of the process of coagulation is uncertain. Fibrin does not exist in the blood as fibrin, but is only formed at the time of coagulation, and is supposed to be formed by some ferment action. (Ferments are bodies capable in small quantity of producing great changes in other bodies without themselves entering into these changes.)

The importance of coagulation is that it checks hæmorrhage, or bleeding, by glueing the cut surfaces together; but for this a small cut might be very dangerous. Blood does not coagulate in health in the vessels during life; but why not, we do not exactly know. Blood, so long as it is in contact with the walls of a living healthy blood-vessel, does not coagulate. But if the wall of the vessel, or the heart, becomes roughened by disease, clots may form obstructing the vessels, and if the clot forms on a valve of the heart, it will impede the proper action of the valve, and will be liable to become detached and swept along to some vessel too small to let it pass through. The result of this is known as embolism, and is serious, owing to the nutrition of the part supplied by the vessel being cut off; and in an important organ such as the brain it is often fatal.

### General Arrangement of the Large Blood-vessels.

(1) From the left ventricle arises the aorta, which, arching over to the left side, goes down along the backbone and divides into two branches for the legs. The aorta gives off the carotid arteries to the head, and the subclavian arteries to the arms. Lower down it gives off large branches supplying the stomach, intestines, and other organs, and finally divides into the two iliac arteries, which supply the pelvis and legs. (2) To the right auricle venous blood from the whole body is returned by two large veins, the superior and inferior vena cava: the former bringing back blood from the head and arms, the latter from the trunk and legs. (3) From the right ventricle the blood goes by the pulmonary arteries to the lungs. (4) To the left auricle the pulmonary veins convey the blood from the lungs.

There are thus two distinct circulations, the greater or systemic, and the lesser or pulmonary—the former carrying blood to and from the general system; the latter carrying it to and from the lungs. Blood in any cavity of the heart in order to get back to the same cavity again must go round both systems, and through all the three other cavities of the heart; for there is no direct passage from one side of the heart to the other. The right side of the heart contains venous blood, the left arterial. The time of a complete circulation is about half a minute.

### Special Circulations.

(1) The heart itself has a special circulation by means of the *coronary* vessels. The coronary arteries arise from the aorta just beyond the semilunar valves and supply the substance of the heart. The coronary veins open directly into the right auricle. (2) The *portal system* is an important special circulation by which blood from the stomach and intestines is taken, not directly into the vena cava, but collected into the portal vein which enters the liver, breaks up into capillaries, and is collected together again by the hepatic vein, which opens into the vena cava below the diaphragm. Hence this blood has two systems of capillaries to pass through; and hence also, which is more important, all food taken up by the bloodvessels from the alimentary canal has to pass through the capillaries of the liver before reaching the heart.

### Proofs of the Circulation of the Blood.

This was proved by Harvey in 1628, before the invention of the microscope, on the following indirect evidence: (1) The valves of the heart and of veins only allow flow of blood in one direction; (2) the blood-vessels of an animal can be injected in one way, but not in the opposite direction; (3) if an artery is ligatured it swells up and pulsates on the side next the heart, the part beyond being pale and empty; (4) if an artery is cut across, blood is pumped from the cut end next the heart, little or none from the other end; (5) if a vein is ligatured it swells up on the side furthest from the heart.

This evidence was confirmed by the direct evidence of Malpighi, who in 1661 demonstrated the capillary circulation under the microscope. If the web of a frog's foot is placed under the microscope the capillaries with the white and red corpuscles circulating in them can be seen.

### Wanted—Nurses.

The difficulty experienced by the heads of private nursing institutions in filling vacancies on their staff appears to become ever greater in proportion as the number of nurses trained every year increases. An instance has occurred within our knowledge where the lady superintendent of a well-managed private nursing home has advertised again and again vainly for suitable nurses, although the salary offered is good and the comfort of the home all that could be desired. Advertisements are not answered, and the lady in question has been at her wits' end in meeting the demands upon her staff. The reason, we fear, is not far to seek, and it is not one which reflects much credit upon the body of nurses in general. The almost universal spirit of restlessness and discontent with anything but what people are pleased to call an "independent position" is rapidly degenerating the nurses of the present day. Every woman who has qualified as a trained nurse wishes either to be in a post of authority and importance in an institution, or if private nursing be her special line, then she must be "independent," and run the risk of being out of work now and again rather than submit to the somewhat greater restraints of working for another, though with the assurance of a certain salary, no responsibility, and the knowledge that if ill she will be well cared for. Is the old motive of helpfulness quite to die out and nursing altogether to lose even the element of self-sacrifice which at first distinguished it? It will be said that nurses have to live, and must consult their own best interests; but on this head, too, they will be well advised in many instances to rest content with the certain earnings secured to them in homes and institutions. The records of the Nurses' Pension Fund would show how far from successful are too many of these "independent" ventures. And in yet another way does this ever-growing restlessness manifest itself, and that is in the shortness of the stay made by the average nurse in any one institution. Not to do the best work that lies in her power, but just to "pick up" a little variety of experience, and have a series of "changes," would seem to be the aim of most. That really good work will ever be done on these lines is impossible.

### Reminiscences of "First Aid."

By a Pupil who Holds a Certificate.

A great many young ladies of the present day appear bitten with the idea that they must know something about nursing, hygiene, and "First Aid." Convinced of the importance of knowledge in emergencies, a course of lectures on "First Aid" to the injured and sick is therefore attended, the syllabus presenting a formidable array of subjects to the uninitiated. Not only are the mysteries of circulation, respiration, digestion, and the names of nerves dealt with, but pupils are instructed how to stop bleeding, to set broken limbs, to pick up insensible ladies or gentlemen in the streets, to throw water over the hysterical, and to restore to life would-be suicides. Notes of the lectures are diligently taken, and certificates of competency are given to the deserving. Many facts (?) brought out at the *vivâ voce* and practical examinations, although preventing the award of a certificate, do not hinder candidates from trying their skill on their neighbours when opportunity arises. A student to prove how thoroughly she had grasped her instructions said that the right "first aid" to render a woman whose dress is on fire consisted in throwing her down on the ground and breaking her leg, to insure her not stirring until all the hearthrugs and greatcoats in the house had been placed over her. Being encouraged by the examiner's questions, she proceeded to say that the leg must be properly set with splints afterwards, and if the patient suffered from "shock" or collapse and was unable to get up and walk about, she might have a little brandy and water, or if a teetotaller a little weak tea or Bovril.

Another candidate was asked to show her treatment of an epileptic fit on a little street arab brought in for the purpose. She gave a description of his imaginary appearance, which was very realistic, and then proceeded to demonstrate that she must get hold of his tongue or he would bite it. She attempted to grasp the unruly member, but the boy, equa-to the occasion, simulated the fit she had described. Howl ever, at last his tongue was grasped, and upon the examiner asking what was to be done with it, she said very decidedly, "Tie it under his chin with an elastic or pin it to his cheek." The boy winced on hearing this, but was reassured when the doctor handed an elastic band. The sight of the boy with his tongue fastened down was impressive. When the writer saw a lad in a real epileptic fit later on, she did not feel tempted to attack his tongue, but simply put the handle of an umbrella between his teeth, to prevent the poor tongue being damaged.

Another candidate was asked what she would do if her husband were brought home with a bad wound in the throat, and seemed likely to die of hæmorrhage. There was no hesitation in her reply that she would bind a triangular bandage firmly round his throat, and tighten it with a tourniquet until the bleeding had stopped. That was the only question the examiner asked, but he intimated his wish to see how she would put on a continuous finger bandage. Five minutes later the finger bandage was pronounced complete; but while the arm was being adjusted in a sling, at a touch the bandage fell in eddying circles at the examiner's feet.

Another lady was asked how the blood circulated in the lower extremities, and what treatment to pursue when a foot was frost-bitten. She replied that the course of the blood was simple; it went down one leg and up the other, and thus "circulated"; that the proper treatment for frost-bite was to burn, boil, or bake the member so as to prevent the mischief spreading, and that to carry this out practically in the best way a tent-bed should be erected close to the kitchen fire, a kettle of water should be boiling on the fire, and a funnel or spout of thick paper should be run from the kettle, so as to carry the steam on to the affected part and thaw the frost out.

Such was the interesting experience of one examination at which the candidates did not all receive certificates.

### Nursing in Rome.

### I.—SANTO SPIRITO HOSPITAL ON THE TIBER.

This is a huge building covering a large expanse of ground. In common with most Italian hospitals, it has a deposit ward, with a small ward for slight accidents and a large one for more serious ones. It is nursed and served by the Sisters of St. Vincent de Paul, and is free for men and children. There is only a small section for women entirely for clinical purposes. The huge deposit ward was once a church, and has a dome elaborately frescoed with stained glass and life-sized figures. The centre portion of the ward is divided off by clear glass partitions. It is lighted by gas and thoroughly and pleasantly warmed. There are plenty of conveniences, with abundance of water laid on. The dispensary, reached by a flight of steps from this ward, has also a frescoed ceiling, and, what is of greater importance to the patients, a quantity of excellent drugs. Everywhere we found stone floors, but the patients are provided with wadded slippers to put on when they get up. Some rich person left money to fit up a small section or ward for chronic cases, and a very pleasant one it is, with the Tiber running past its windows and the Italian sunshine flooding every corner. The operating theatre also looks out on the river, and is lined and floored with thick white ground glass, which is easily cleaned. On the walls are fixed brackets holding barrels of disinfectants with tubes leading down and on to the operating table. Water is also laid on, and several small rooms leading out of it contain every medical and surgical appliance that can possibly be needed. The beds are clean and good, the nightgowns white, and no unpleasant smells anywhere. There are three doctors to each section, and they visit the patients twice a day. Bright tin dishes are used for carrying the food from the kitchen to the wards. In one little room a monk waits in case of any dying patient requiring his services. There is a ward for consumption, and isolated rooms for fever and small-pox, but they do not isolate typhoid. This hospital occupies both sides of the road, and, crossing over, we came to a huge vaulted ward with three aisles in it.

The doctors' private rooms are very inferior, and so small and dark that they cannot live in them. The Palace of Santo Spirito contains a vast store house for drugs, bandages, &c., which supplies all the hospitals in Rome, and we saw a cart-load of gauze bandages starting off for distribution. This immense building also contains a foundling hospital, in which there are 1,510 children. The wards are full of pretty cots and wee babies in the charge of sisters and a number of nurses, and all were evidently well cared for. There is a large drying ground, and the laundry is worked by the best and newest machinery; 4,000 pieces of linen are washed daily. We were conducted over this interesting hospital by Dr. Raffaele Bastianelli.

### Novelties for Nurses.

### WARD AND SICK-ROOM SHOES.

A short time ago nurses experienced some difficulty in obtaining suitable shoes for hospital wear. But the makers of shoes have found out that the large army of nurses have special requirements, and now the difficulty is to choose between the many not the few. One thing is very certain, and that is that it is far less in the long run to buy really good articles, and quality, not cheapness, is mostly sought for. Messrs. Garrould, recognising this fact, have taken pains to secure a really durable and good ward shoe for their customers. The shoes are guaranteed of real morocco, are hand sewn, and heeled with a durable rubber, to last as long as the shoes. The toes are not pointed, and the tread is wide. They are finished by a neat bow of leather, and represent a neat and workmanlike appearance.

### On the Choice of Pupils for Professional Nursing.

FROM AN OLD-FASHIONED STANDPOINT.

By Miss A. L. PRINGLE, late Matron of St. Thomas's Hospital, and Superintendent of the Nightingale School, etc.

THE English historian of the latter half of this century, contemplating the social life of the people, will observe that the stress of circumstances in these days has thrown upon a large proportion of the women the duty of maintaining themselves. It is not enough now that they work industriously at home to save money; they must go out and earn it. In an old novel—"Deerbrook"—Miss Martineau brings her heroine to the strait of adopting one of these alternatives, and causes her to choose dispensing with servants and remaining at home, bringing out with a loving insight the reasons of its preferableness. In such a strait now the heroine would certainly make a different choice. Nay, our girls do not wait for a financial crisis in the home; it is almost the rule for them to have a regular calling. They have taken the labour market by storm, and now find they must accept the conditions of the rights they have attained. In former days, when a woman's usual life lay in the sacred dependence of home, if misfortune drove her out into the world, she would seek a situation as governess, or open a little school of her own, or obtain the position of local postmistress, all unrestrained by any call for certificates of training. Work in public offices was hardly thought of for women, any more than professional nursing or many of the other occupations now in vogue. To-day a woman who has stayed at home to render the services claimed of her there, if at length thrown on the world to earn her living, will find hardly any space for an untrained worker, and that her applications for training will in most cases be declined on the ground of her being over the usual age.

Now for many callings there is good reason for fixing an early age for training. Youth is more apt for learning, and the knowledge required can best be given in the profession itself. For the worker, too, as a worker, it is an advantage, though, in the case of a woman, it carries with it the loss of opportunities of learning those domestic duties which will devolve on her later should she quit her profession for a home of her own. More than this, we have all seen cases where the public work has created a positive distaste for home life on the old safe lines, and has led to a lamentable perversion of character. But we will not dwell on this now. At first sight it might seem that early age was a matter of course for professional training in all cases, but on examination we perceive that for at least one very widely followed occupation—that of nursing—immaturity is not a serviceable condition.

It is obvious to all that we have at once too many nurses and not enough of good ones. Is there an error in the selection in some quarters? The training ground is very limited, and it is desirable to use it as fruitfully as possible.

As to there being too many trained nurses, we will not say there are many without employment, but that it has become the fashion to use them where they are not required. In hospitals they are certainly necessary; there is no room there for the amateur. To a large extent, also, they are necessary in systematic work in the homes of the poor; and they are required in other ranks in cases of special difficulty, or where no competent and trustworthy relative or servant is at hand; but it is an abuse to employ them without such necessity. Do not let us hustle Miss Ophelia and Uncle Tom off the scene to give Eva a professional nurse. The expense, too, is often an intolerable addition to the burdens of illness.

Regarding the selection of women for this work one may safely say it is a greater matter than the method of their professional training. A particular character and aptitude are necessary, and a ground work of knowledge and experience must be brought as a dowry to the work if the technical training is not to be idle or mischievous.

To be a good nurse it is necessary to have a practical acquaintance with the laws of health. Nursing means the observance of these, as well as a light touch, calm movements, and an aptitude for learning quickly how to apply the remedies ordered. It means insight and observation, sympathy and tact, prudence and patience, and an experience of life which gives the clue to many symptoms, and aids in the treatment of the case. A nurse possessing these qualifications gains the confidence of doctor and friends, so that all pull together for the end in view. They are necessary in all nurses, except that a wide experience of life may well be dispensed with in the case of a young girl nursing in her own home. They are sufficient in the greater number of cases nursed in private life, but they need to be trained and supplemented in the professional nurse, whether her occupation lies in the hospital or elsewhere. The management of a ward requires methods which must be taught to beginners. The medical school, too, gives duties to the hospital nurse, and she has to learn many things for the furtherance of that which bear only indirectly on the patient's cure. She must make diligent use of her opportunities in hospital to fit herself for attendance on every description of case, and she must adapt herself intelligently to the division of labour which obtains in these huge households. She comes into the hospital to serve it in its own needs, and to gain from it that acquaintance with disease and its treatment without which, however well she might manage stray cases in her own home, she would be awkward, unready, and sometimes useless in the sudden calls of a professional nurse's life. But she must bring to the work those aptitudes and qualifications of head, heart, and hand with which we set out. They are the growth of thoughtful years spent in common life. Take a girl of twenty, with capabilities of all we desire for this work. Take the same girl at twenty-seven, stipulating that she shall have responded well to the graces of experience and discipline given her in that time. Which is the more fit to come into the public life of the hospital with its sad secrets, its exhausting demands on mind and body, its searching tests of character? Some will say, "Let us have the young girl fresh from school, having still the habits of study; she passes the examination betters than the older ones." It is true she does that. But she is wanting in the deeper preparation for the work. And it is a want which can never be supplied in THE HOSPITAL. It is out of a sufficient experience of ordinary life that her usefulness in the extraordinary will be drawn. The case is different from that of, for instance, a school teacher, whose home life continues throughout her scholastic training.

The objection will be made that a girl must choose her work in her teens, and that it is putting the work and worker both at a disadvantage thus to defer the beginning. But those who have followed our course of thought so far will see their way out of that difficulty.

The proper care of the sick requires far more than professional training alone can give, and the conditions of work in our public hospitals make severe demands of their own. In the nurse employed there you want the same qualities that would make a good mother and mistress of a large family in arduous times. Nor do you want those qualities inherent only; as we have already shown they must have attained a fair growth in the nursery of private life before being transplanted to the hospital.

For twenty-five vacancies a superintendent has, perhaps five hundred applications. It needs great judgment to make a proper selection. However, she will find that a very large proportion of candidates will show no particular fitness, and that some have an evident unfitness. Nature is not wasteful.

A great many would do better in other callings. Let them go without delay to the desk, the needle, or the school-room. In the reduced list, besides a few eligible candidates, the practised eye will discern here and there a girl of particular promise still some years under the desired age. This one is not to be sent adrift. She is to have matters a little explained to her, and to be advised to occupy herself for some years in the atmosphere of private life in some employment that will be educative for her future calling—that of governess, for instance, or mother's help, or companion to some invalid who needs help in her housekeeping. It is not good to spend those intervening years in a small institution. She would still miss the experience of common life, and would come to the beginning of her hospital work fagged and half worn out. Besides, those small institutions need continued and whole-hearted service. It would almost be better to begin the hospital work itself prematurely. Indeed, though very sparingly, the superintendent may indulge herself by occasionally taking one of these "babies" on her staff. There are circumstances that justify it for the worker, and her deficiencies will be made up by the seniors whose lives she helps to brighten. In rare cases it answers surpassingly well. But experience shows that these exceptional instances are rare, and that, commonly, early admissions weaken the working power of the staff and lead to early exhaustion of the worker.

How then are we to discover her whom we are seeking? She will be distinguished in almost all cases as having been the useful daughter at home, the mother's right hand, the family nurse. Necessities find out the willing and capable; favouritism gives privileges, not tasks. In most families there is one whose helpfulness has kept her at home while the others one by one have gone their way; a time comes when she finds herself at last free from home duties, perhaps also bereft of home ties and of means. Well for the hospital that opens wide its doors with welcome to these. They are nature's provision for its needs. Come, reap your harvest dear home daughters, the harvest most acceptable to such as you. You do not want amusements, riches, leisure; you want a new task to take the place of the precious duties now done; you want labour and interest, an outlet for your energies and affections, scope for the influence your previous life has given you.

It is true such workers are not to be had in abundance, but it is not necessary the whole staff should be composed of them. If they are the type and preponderating element they will impart their spirit to a minority of a different stamp, whose varied gifts can thus safely be gathered up into the treasury of nursing power.

Do any ask from what class these workers should be drawn? There is room on a hospital staff for many degrees. Character, capacity, kindness you must have, and true refinement and dignity; all this is not found too often in any class. Value it, therefore, wherever you find it, and cherish it by every means in your power. Later you can determine the respective appointments by special fitnesses.

Methods of training and systems of work may well vary. The most careful efforts may be expected to produce less than perfection. But in order that any method may succeed and not rather do lamentable mischief, it is necessary that those at the helm should have a clear idea of the work, and should choose their nurses always in view of their high and difficult vocation, to be, at the risk of their own lives, the efficient dependence of the sick, the sorrowful, and the dying.

A. L. P.

### "The Hospital" Convalescent Fund.

### THE NURSES' BED.

The hon. secretaries beg to acknowledge the receipt of 10s. from Nurses Jennings and Mason; 2s. 6d. from Miss Theresa Steer.

### The Book World for Women and Nurses.

[We invite Correspondence, Criticism, Enquiries, and Notes on Books likely to interest Women and Nurses. Address, Editor, The Hospital (Nurses' Book World), 428, Strand, W.C.]

The Cult of Beauty: A Handbook of Personal Hygiene. By C. J. S. Thompson. (London: Walter Scott, Limited, Paternoster Square. Price 2s. 6d.)

This is a well-written and daintily got-up volume on a subject of perennial interest. While it has no pretensions to originality, it commends itself on the ground of moderation; for the author neither wholly condemns nor rashly advises the use of those aids to beauty which, openly or surreptitiously, a very large number of women indulge in. A number of prescriptions for face, hands, teeth, and hair are inserted in the volume, and while many of them are distinctly useful, all are harmless, and the author is careful to make it evident that cosmetics and their kin will never take the place of hygienic treatment. His remarks on the bath are very sensible. The chapters on ears and noses are more interesting as a matter of curiosity than practically valuable, and the indications of character read from the shape of the nose, though not wholly meaningless, tend more or less to that bourne where phrenology and chiromancy dwell together in pseudo-scientific union.

Physical Culture for Men, Women, and Children. (Ling-Swedish System.) By Arthur Edmund Tanner. (Simpkin, Marshall, Hamilton, Kent, and Co., Limited. Price 2s.)

It may be taken as a certainty that the importance of physical culture has been so universally recognised that we venture to hope it is not necessary to repeat the well-known reasons as to why some kind of daily physical exercise is advisable. The largely increasing number of manuals on the subject bear witness to the increasing demand for such aids, and to the fact that there is a "something" in the practice of the various systems which has already proved beneficial, else the demand for these books would cease. It is well known that although one system may admirably suit some it will not succeed with others. "*Quot homines, tot sententiæ.*" Now Dr. Tanner's book seems to cover a wide area, and supplies directions for an easy course of movements which may be practised with equal advantage by men, women, or children; at the same time more advanced exercises are added for athletes. The general course is so carefully chosen that, taken in the order given, one movement serves as an actual rest and relief to the muscles used in the foregoing exercise, and this naturally tends to dispel any fatigue which might reasonably be expected from the continuous series of movements, and it will be found that after the first or second day the result of the course will be increased energy and freshness, and a system braced for the day's work. The time allotted to this health-giving practice is so short that even the busiest among us will find it within the limits of possibility, and will never regret any extra effort made to fit in their daily exercise. To this class of people especially is recommended the movements which seem at first sight so slight and trivial, but which serve as the best correctives for the faulty postures which are unavoidably acquired by all engaged in sedentary employments. While this training is undoubtedly followed by the best results when tried by the young, we must bear in mind that "it is never too late to mend," and an easy but progressive course of exercise such as Dr. Tanner now offers us will be more effectual in restoring and preserving health of body and mind than unlimited doses of physic, and a regular observance of these rules will soon banish a host of enemies, such as indigestion and its attendant complaints, while the whole carriage will gain in vigour, dignity, and grace.

### For Reading to the Sick.

LOOKING BACK.

**Motto.**

What hath been bringeth what shall be.—*E. Arnold.*

**Verses.**

The year departs ! A blessing on its head !
We mourn not for it, for it is not dead ;

The passing breezes gone as soon as felt,
The flakes of snow that in the soft air melt,
The smile that sinks into a maiden's eye,
They come, they go, they change, they do not die.
So the old year—that fond and formal name—
Is with us yet—another, and the same.
—*H. Coleridge.*

Mark how there still has run, enwoven from above,
Thro' thy life's darkest woofs, the golden thread of love.
—*Trench.*

Have I laid by from summer hours,
Ripe fruit as well as leaves and flowers?
Hath my past year a growth to harden,
As well as fewer sins to pardon?
Is God in all things more and more
A King within me than before? —*Faber.*

If thou would'st reap in love,
First sow in holy fear ;
So life a winter's morn may prove
To a bright endless year. —*Keble.*

**Reading.**

A simile of life. He is first a man when he comes to a certain steady use of reason . . . and when that is, ali the world of men cannot tell precisely. Some are called of age at fourteen; some at one-and-twenty ; some never ; but all men late enough—for the life of a man comes upon him slowly and insensibly. But as, when the sun approaches towards the gates of the morning, he first opens a little eye of heaven, and sends away the spirits of darkness, and gives light to a cock, and calls up the lark to matins and peeps over the eastern hills, thrusting out his golden horns . . . . and still while a man tells the story, the sun gets higher and higher, till he shows a fair face and a full light, and then he shines one whole day, under a cloud often, and sometimes weeping great and little showers; and sets quickly; so is a man's reason and his life.—*Jeremy Taylor.*

A mist before and a mist behind, we stand in the midst, and see not our way. God's mercies surround us on every side, but until they are past we mark them not. In our blindness we struggle against that which memory will show in after years to have been our greatest good. Oh! for a trusting heart; to shut our eyes and hold His hand; and tread unshrinkingly the darkened way. The steeper the hill the firmer the grasp. Look up, look up, and never stay thy feet. Onward and upward until the golden gate is reached. —*Heart Musings.*

### Appointments.

[It is requested that successful candidates will send a copy of their applications and testimonials, with date of election, to The Editor, The Lodge, Porchester Square, W.]

Batley and District Cottage Hospital.—Miss Emma Cann has been elected Matron at this hospital. She was trained at the South Devon Hospital, Plymouth, held the post of sister there, and that of senior sister at the Hospital for Women, Soho Square, which hospital she is leaving for her present appointment.

### Everybody's Opinion.

[Correspondence on all subjects is invited, but we cannot in any way be responsible for the opinions expressed by our correspondents. No communications can be entertained if the name and address of the correspondent is not given, or unless one side of the paper only be written on.]

ROYAL NATIONAL PENSION FUND FOR NURSES.

The secretary of the Royal National Pension Fund for Nurses having forwarded the following letter to us, we have pleasure in publishing it, since we gather that the writer wishes to aid her fellow nurses by presenting to them a practical example of the benefits of providing for sickness by becoming a member of the Pension Fund.

"Having now recovered sufficiently to be able to resume work, I wish to say how deeply grateful I am to the Committee of the Sick Pay Fund for the generous treatment I have met with for the past year. The weekly allowance has greatly aided my restoration to health, by enabling me to stay in a suitable climate for many months.—Yours very truly, M. A. Townshend."

It may interest our readers to know that the writer of the above letter has received sick pay since September 11th, 1894, amounting altogether to £51, and that she has paid in premiums only £1 13s.—Ed. *T. H.*

### Where to Go.

Ancient Glass and China.—At the Royal Aquarium, on September 10th, an exhibition will commence of old and modern china and glass. Amongst the exhibits will be vases from the Emperor's palace at Pekin—which once belonged to General Gordon—and a number of Nelson relics will be shown also.

### Notes and Queries.

The contents of the Editor's Letter-box have now reached such unwieldy proportions that it has become necessary to establish a hard and fast rule regarding Answers to Correspondents. In future, all questions requiring replies will continue to be answered in this column without any fee. If an answer is required by letter, a fee of half-a-crown must be enclosed with the note containing the enquiry. We are always pleased to help our numerous correspondents to the fullest extent, and we can trust them to sympathise in the overwhelming amount of writing which makes the new rules a necessity. Every communication must be accompanied by the writer's name and address, otherwise it will receive no attention.

**Queries.**

(240) *Guild of St. Barnabas.*—How can I become a member of the "St. Barnabas' Guild"?—*Nurse F.*

(241) *District Nurse.*—Is there an institution in Birmingham or Manchester where the nurses are paid a fee per visit to the poor?—*S. G.*

(242) *Isinglass.*—Please tell me the best way to use isinglass when ordered as an extra "nutritive" in all drinks either warm or cold.—*M.R.*

(243) *Probationer (R.)*—You will find particulars of nurse training schools in "How to Become a Nurse," by Honner Morten, The Scientific Press, 428, Strand. A certificate of one year's training would carry no weight.

(244) *Male Nurse.*—What is the training necessary for the fully-qualified male nurse (including cookery and massage), and where may that training best be obtained?—*A. G. T.*

**Answers.**

(240) *Guild of St. Barnabas (Nurse F.)*—Write to the editor of the paper of the Guild "Misericordia" at W. Knott, 26, Brooke Street, Holborn.

(241) *District Nurse* (S. G.)—We do not think such an arrangement is made. The Manchester and Salford Sick Poor Nursing Institution and Birmingham District Nursing Society might be applied to. Also see Burdett's "Hospital and Charities Annual."

(242) *Isinglass (M. R.).*—Isinglass should be soaked in a small quantity of water for about one hour, and then transferred to an enamelled saucepan and stirred over a slow fire until melted, then added to the drink, hot or cold as required.

(243) *Probationer.*—Could you tell me of one of two good provincial hospitals where laoies are taken for a year's training and certificate given for a premium from £20 to £30 per annum? The North preferred, but not essential.—*R.*

(244) *Male Nurse (A. G. T.)*—Unfortunately, as we have frequently stated in this paper, no institution trains nurses in this country, consequently there is no standard of qualification. It is hoped that some hospital will eventually be induced to train male nurses. Applications should be made to the Seamen's Hospital, Greenwich, by those who desire to train as male nurses.

